# Human *Streptococcus agalactiae* Isolate in Nile Tilapia (*Oreochromis niloticus*) 

**DOI:** 10.3201/eid1505.080222

**Published:** 2009-05

**Authors:** Joyce J. Evans, Phillip H. Klesius, David J. Pasnik, John F. Bohnsack

**Affiliations:** US Department of Agriculture, Chestertown, Maryland, USA (J.J. Evans, D.J. Pasnik); US Department of Agriculture, Auburn, Alabama, USA (P.H. Klesius); University of Utah Health Sciences Center, Salt Lake City, Utah, USA (J.F. Bohnsack)

**Keywords:** Zoonoses, streptococci, Streptococcus agalactiae, group B streptococci, fish, Nile tilapia, Oreochromis niloticus, human, infectivity, dispatch

## Abstract

*Streptococcus agalactiae*, the Lancefield group B streptococcus (GBS) long recognized as a mammalian pathogen, is an emerging concern with regard to fish. We show that a GBS serotype Ia multilocus sequence type ST-7 isolate from a clinical case of human neonatal meningitis caused disease and death in Nile tilapia (*Oreochromis niloticus*).

*Streptococcus agalactiae*, group B streptococcus (GBS), has a broad host range and is pathogenic to mammals, reptiles, amphibians, and fish (*1*). This organism has also been identified in aquatic mammals, both captive and wild bottlenose dolphins (*Tursiops truncatus*) (*2*,*3*). GBS causes mastitis in cattle and meningitis in human neonates (*4*). It also causes meningoencephalitis in fish (*5*,*6*). Since the first report of GBS in hatchery-reared freshwater fish in the United States in 1966 (*7*), reports of piscine GBS have increased. Piscine GBS, like human and bovine GBS, is found worldwide and affects a variety of freshwater and marine fish under a broad spectrum of environmental conditions (*6*,*8*).

Sustained emergence of human GBS neonatal disease for undetermined reasons has spurred many GBS genomic diversity studies. Acquisition of bovine GBS by humans has been proposed as 1 plausible explanation (*9*). Human and bovine GBS isolates have been considered genetically distinct populations (*10*) and as related populations arising from a common ancestor, presumably bovine (*1*,*9*).

Two lineages of bovine GBS (multilocus sequence type ST-23 and ST-61) appear to have a genetic relationship with human GBS. Human serotype Ia strains in the ST-23 lineage and human serotype III ST-17 strains are related to bovine ST-61 strains and frequently associated with neonatal infections (*10*,*11*). Phylogenetic studies have focused on homogeneity or heterogeneity of lineages and association with carried or invasive GBS. Many researchers believe that GBS serotype does not correlate with evolutionary relationships. Strains of different serotypes and sequence types often share more genes than strains of the same serotype and contain carried and invasive strains, traits suggestive of opportunistic pathogenicity (*9*,*11*,*12*).

Whether GBS is a zoonotic organism has not been adequately explored. Conclusions of genomic studies can only infer virulence, infectivity, and transfer between different animals on the basis of serologic, molecular, and computational analyses of GBS isolates. Bovine GBS isolates of unknown sequence type were not infective for Nile tilapia (*Oreochromis niloticus*) (*13*). However, potential for infection between homothermic hosts and poikilothermic animals has been demonstrated.

GBS transmission from mice to reptiles occurs by oral ingestion (*14*). In tilapia (mean weight 40 g) infectivity studies with a dolphin GBS isolate, disease signs and a mortality rate of 90% were noted within 6 days postinoculation with 10^7^ CFU/fish (*3*). Piscine, dolphin, and human GBS isolates have been reported (*15*) to share the same serotype (Ia) and sequence type (ST-7) as that reported from human GBS carried (strain from a person with no evidence of disease) and neonatal invasive strains from Japan (*4*) and North America (isolate A909) (*11*). This finding indicates that piscine and dolphin GBS isolates may have been derived from human sources and caused a fish epidemic in Kuwait and that serotype Ia ST-7 GBS may cause a zoonosis (*15*). Non–ST-7 fish isolates are widely divergent from other animal GBS isolates (*12*,*15*). We conducted a study of experimentally induced infection to determine whether a human serotype, Ia ST-7 GBS isolate, could cause disease signs and death in fish.

## The Study

Nile tilapia served as experimental fish because they could be held at warm water temperatures closer to the normal human body temperature of 37°C than other available fish species. Seven groups of 10 tilapia (mean ± SE weight 28.20 ± 0.51 g) each were housed in 57-L aquariums at the Aquatic Animal Health Research Laboratory (Chestertown, MD, USA). All tanks were supplied with flow-through dechlorinated tap water and 2 submersible heaters and air stones to maintain desired water temperature and dissolved oxygen (DO) levels. Water quality (mean ± SE temperature, DO, and ammonia concentration) was measured daily by using a YSI 85 meter (Yellow Springs Instrument Co., Yellow Springs, OH, USA) and a Fresh Water Aquaculture Kit (Model AG-2; LaMotte Company, Chestertown, MD, USA). Temperature was 32.1 ± 0.09°C, DO was 4.2 ± 0.14 mg/L, and ammonia concentration was 0.74 ± 0.08 mg/L. Fish were fed daily (4% of bodyweight) with Aquamax Grower 400 fish feed (Purina, Brentwood, MO, USA) and maintained and handled according to Institutional Animal Care and Use Committee–approved guidelines.

The serotype Ia ST-7 human GBS isolate (ID# 510012) was obtained from a patient in Japan who had neonatal meningitis. The isolate was cultured overnight on 5% sheep blood agar (SBA; Remel, Lenexa, KS, USA) at 32°C. Before the study, the isolate was passed through 5 Nile tilapia (weight 18.4 ± 0.48 g) 1 time each by intraperitoneal injection of 10^7^ CFU of GBS/fish. Specimens from a fish that died 3 days postchallenge were cultured on SBA, and GBS was recovered from nostrils, intestines, posterior kidney, and brain. One GBS colony isolated from brain was cultured on SBA, typed as GBS by Lancefield grouping (*6*), and used for experimental infection.

Serial dilutions of the GBS isolate were prepared in tryptic soy broth (TSB; Remel), and 10 fish (weight 28.2 ± 0.51 g) were each injected intraperitoneally with 0.1 mL of inoculum at 10^7^, 10^6^, 10^5^, 10^4^, 10^3^, or 10^2^ CFU/fish. Ten control fish were injected with 0.1 mL of TSB only. Fish were placed in separate 57-L aquariums at 32°C according to dose and monitored daily for signs of disease and death for 14 days postchallenge. Moribund fish were humanely euthanized by an overdose of tricaine methanesulfonate (MS-222; Argent Chemical Laboratories, Redmond, WA, USA).

Bacterial samples were obtained from nostrils and brains of all moribund or dead fish and cultured on SBA at 32°C for 24 h for GBS. Identification of GBS was performed by using methods of Evans et al. (*6*) and the BIOLOG MicroLog3 Microbial Identification System (BIOLOG, Hayward, CA, USA) according to the manufacturer’s instructions. BIOLOG results were compared with a Microlog database (www.biolog.com/mID_product.html); a similarity index >0.50 and high probability (>90%) were considered a strong confirmation for GBS.

Within 7 days postchallenge, the 10^2^, 10^3^, and 10^7^ CFU/fish groups had a mean cumulative mortality rate of 11.7% (7/60) (Figure). Overall, deaths occurred on day 2 and the mortality rate reached 20% after 14 days of observation. Deaths after day 10 occurred in fish that received 10^6^ CFU. Sampled organs were negative for GBS and deaths were attributed to tank mate aggression to weakened fish. A linear dose response was not seen. Deaths occurred at low (10^2^–10^3^ CFU/fish) and high (10^7^ CFU/fish) doses but not at median doses (Table). Disease signs in tilapia exposed to human GBS were lethargy, anorexia, dark coloration, opaque eyes, and remaining stationary at the bottom of the tank.

**Figure:**
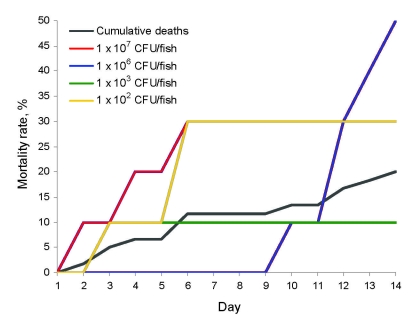
Mortality rates for 60 Nile tilapia at all doses (black line) and 10 tilapia each challenged with a human *Streptococcus agalactiae* isolate (#510012): 10^2^ (gray line), 10^3^ (green line), 10^6^ (red line), and 10^7^ (blue line) CFU/fish. No deaths occurred at 10^4^ and 10^5^ CFU/fish or in tryptic soy broth controls.

**Table:** Mortality rates among Nile tilapia (*Oreochromis niloticus*) infected with Streptococcus agalactiae*

Dose (CFU/fish)	No. (%)† deaths
Tryptic soy broth (control)	0^a^
10^2^	3 (30)^ab^
10^3^	1 (10)^a^
10^4^	0^a^
10^5^	0^a^
10^6^	5 (50)^b^
10^7^	3 (30)^ab^

*Ten fish per dose group were intraperitoneally injected with human *S*. *agalactiae*/fish or tryptic soy broth and observed for 14 days postchallenge at a water temperature of 32°C. †Superscript letters indicate significant differences (p<0.05) in percentage mortality rates between groups, determined by using the SAS program (SAS Institute, Cary, NC, USA) lifetest procedure (Kaplan-Meier method).

All sampled organs from fish dying within 7 days of infection contained β-hemolytic, gram-positive, oxidase-negative, catalase-negative bacteria. BIOLOG analysis confirmed identification as GBS (similarity index 0.79, probability 100%). None of the TBS-injected control fish showed signs of disease or died.

## Conclusions

Deaths among experimentally infected Nile tilapia indicate that an Ia ST-7 human GBS isolate can be pathogenic to fish. Such isolates have been associated with human (*4*,*12*) and fish disease as well as with a marine mammal sampled during a GBS fish kill (*15*). The human isolate was virulent in tilapia at 10^2^, 10^3^, 10^6^, and 10^7^ CFU/fish, and GBS was reisolated from diseased fish. These experimental findings suggest GBS transmission between mammals and fish and that GBS-induced fish epidemics can originate from mammalian sources. Although we studied only 1 human isolate, other isolates or isolates repeatedly passed through fish may be more virulent. Susceptibility to GBS may also be enhanced by suboptimal environmental conditions, such as low DO, high ammonia levels, euthrophication, harmful algae, and changing or extreme water temperatures (*5*,*6*). Future histopathologic investigations may characterize the distribution and nature of the host response to human GBS.
